# Editorial: The genetic intersection of mental and physical health: unraveling shared heritable risk factors

**DOI:** 10.3389/fpsyt.2026.1930178

**Published:** 2026-08-04

**Authors:** Hongbao Cao, Sha Liu, Shaolei Teng

**Affiliations:** 1 School of Systems Biology, George Mason University, Fairfax, VA, United States; 2 Department of Psychiatry, First Hospital/First Clinical Medical College of Shanxi Medical University, Taiyuan, China; 3 Department of Biology, Howard University, Washington, DC, United States

**Keywords:** gene–environment interaction, Mendelian randomization, mental health, neuroimaging, physical health, precision medicine, psychiatric genetics, shared heritability

## Background and aims

Mental and physical disorders have long been investigated as separate clinical entities. Increasing evidence from genetics, transcriptomics, neuroimaging, and multi-omics studies demonstrates that psychiatric disorders and systemic diseases frequently share heritable risk factors and biological pathways (1, 2). Immune dysregulation, mitochondrial dysfunction, metabolic remodeling, and gene–environment interactions have emerged as common mechanisms that transcend traditional disease boundaries (3). Motivated by these advances, this Research Topic was established to provide a multidisciplinary platform for investigating the genetic intersection between mental and physical health.

The twelve articles collected in this Topic span original research, reviews, systematic reviews, and conceptual analyses, illustrating how modern systems biology approaches are reshaping our understanding of psychiatric and physical comorbidity. Rather than treating mental disorders as isolated brain diseases, the contributions collectively support a framework in which common genetic architecture interacts with environmental exposures to produce diverse clinical phenotypes.

## From association to shared biology

Several studies reveal convergent biological mechanisms linking mental and physical disorders. Beaulieu Perez et al. explored inflammatory biomarkers associated with depression in women with diabetes, emphasizing immune regulation as a shared pathway. Yan et al. identified common genes shared by pulmonary arterial hypertension and major depressive disorder. Huang et al. (4) investigated the bidirectional molecular relationship between anxiety and COVID-19, while Chen et al. highlighted mitochondrial and aging-related biomarkers in major depressive disorder. Collectively these studies support immune, inflammatory, and metabolic dysregulation as common biological foundations of mental–physical comorbidity.

## From shared biology to causal mechanisms

Yang et al. characterized abnormal cerebral–limbic functional connectivity distinguishing bipolar mania and bipolar depression. Liu et al. identified age-dependent gray matter volume alterations in healthy siblings of schizophrenia patients, suggesting inherited neuroanatomical endophenotypes. Tu et al. integrated transcriptomic decoding with cerebral blood flow abnormalities in first-episode major depressive disorder. Zhou et al. combined Mendelian randomization and immune transcriptomics to investigate depression and cardiovascular disease. Kouris and Kumsta reviewed polygenic scores in stress-related psychopathology, whereas Abualia et al. discussed endocrine-sensitive gene–environment interactions through the RE-MEND framework. Together these studies illustrate the transition from association toward causality and systems-level understanding.

## From mechanisms to precision medicine

Cui et al. demonstrated that depression and cognition mediate the relationship between REM sleep behavior disorder and activities of daily living in Parkinson’s disease. Zeng systematically reviewed suicide risk among patients with heart failure, emphasizing the need for integrated psychiatric care. Together with Beaulieu Perez et al., these findings indicate that psychiatric symptoms are integral components of chronic disease progression and should be incorporated into precision prevention and individualized management strategies.

## Future perspectives

**Figure 1:**
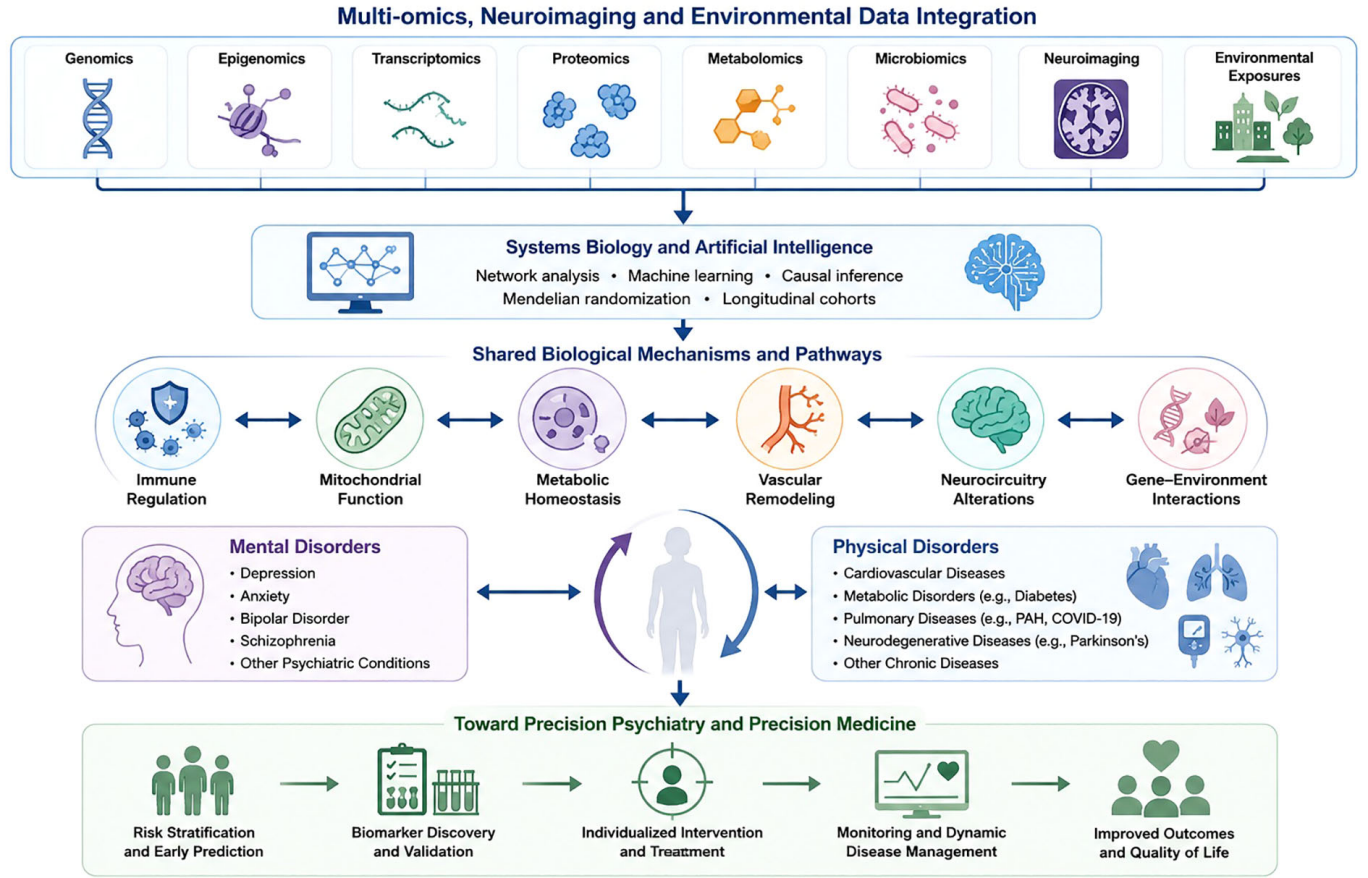
Integrative framework for precision psychiatry and medicine. Multi-omics, neuroimaging, and environmental data are integrated using AI and systems biology to identify shared disease networks and high-risk individuals.

The collective contributions illustrate a conceptual shift from studying isolated diseases toward understanding interconnected biological systems. Future integration of genomics, epigenomics, transcriptomics, proteomics, metabolomics, microbiomics, neuroimaging, and environmental exposure data, coupled with artificial intelligence and systems biology approaches, will accelerate identification of shared regulatory networks and high-risk individuals (see **Figure 1**). Such interdisciplinary efforts are expected to redefine disease classification and facilitate precision psychiatry and precision medicine.

Taken together, the studies in this Research Topic demonstrate that shared heritable factors provide a unifying framework for understanding the intersection of mental and physical health, while also opening new opportunities for mechanism-based diagnosis and therapeutic intervention.
